# Using the capture-recapture method to estimate the human immunodeficiency virus-positive population

**DOI:** 10.4178/epih.e2017042

**Published:** 2017-10-10

**Authors:** Jalal Poorolajal, Younes Mohammadi, Farzad Farzinara

**Affiliations:** 1 Department of Epidemiology, School of Public Health, Hamadan University of Medical Sciences, Hamadan, Iran; 2 Research Center for Health Sciences, School of Public Health, Hamadan University of Medical Sciences, Hamadan, Iran; 3 Modeling of Noncommunicable Diseases Research Center, School of Public Health, Hamadan University of Medical Sciences, Hamadan, Iran; 4 Social Determinants of Health Research Center, School of Public Health, Hamadan University of Medical Sciences, Hamadan, Iran

**Keywords:** Capture-recapture method, HIV seropositivity, Linear models, Iran

## Abstract

**OBJECTIVES:**

The capture-recapture method was applied to estimate the number of human immunodeficiency virus (HIV)-positive individuals not registered with any data sources.

**METHODS:**

This cross-sectional study was conducted in Lorestan Province, in the west of Iran, in 2016. Three incomplete sources of HIV-positive individuals, with partially overlapping data, were used, including: (a) transfusion center, (b) volunteer counseling and testing centers (VCTCs), and (c) prison. The 3-source capture-recapture method, using a log-linear model, was applied for data analysis. The Akaike information criterion and the Bayesian information criterion were used for model selection.

**RESULTS:**

Of the 2,456 HIV-positive patients registered in these 3 data sources, 1,175 (47.8%) were identified in transfusion center, 867 (35.3%) in VCTCs, and 414 (16.8%) in prison. After the exclusion of duplicate entries, 2,281 HIV-positive patients remained. Based on the capture-recapture method, 14,868 (95% confidence interval, 9,923 to 23,427) HIV-positive individuals were not identified in any of the registries. Therefore, the real number of HIV-positive individuals was estimated to be 17,149, and the overall completeness of the 3 registries was estimated to be around 13.3%.

**CONCLUSIONS:**

Based on capture-recapture estimates, a huge number of HIV-positive individuals are not registered with any of the provincial data sources. This is an urgent message for policymakers who plan and provide health care services for HIV-positive patients. Although the capture-recapture method is a useful statistical approach for estimating unknown populations, due to the assumptions and limitations of the method, the population size may be overestimated as it seems possible in our results.

## INTRODUCTION

Measuring and understanding the size of the human immunodeficiency virus (HIV)-positive population is a critical public health challenge. Estimates of population size are required to help make decisions about how resources should be efficiently allocated to the planning and management of programs targeting HIV. Without an accurate estimate of the magnitude of the HIV-positive population, it is impossible for countries to plan and implement HIV prevention, care, and treatment programs [1].

Several methods have been suggested for estimating hard-to-count populations, including the network scale-up method [2], the census and enumeration method [3], and the capture-recapture method [4]. The capture-recapture method is widely used in ecology to estimate the unknown size of populations of wild animals [5]. However, the capture-recapture method can be applied to any situation with 2 or even more incomplete lists [6]. This method has been recently used in epidemiologic studies for estimating hidden populations with a particular disease and assessing the completeness of ascertainment of disease registers [7-10].

The purpose of this study was to estimate the number of HIV-positive individuals to facilitate planning about the provision of health services for this population. Until reliable information about the size of the HIV-positive population is available, it is difficult to design effective measures and to provide health care services to this population. Therefore, this study was conducted to estimate the size of the population living with HIV/acquired immunodeficiency syndrome (AIDS) who are neither diagnosed nor registered with any of the available data sources.

## MATERIALS AND METHODS

This registry-based cross-sectional study was conducted in Lorestan Province, in the west of the Islamic Republic of Iran (hereafter Iran), in 2016. The Ethics Committee of Hamadan University of Medical Sciences approved the study. Data on HIV-positive patients were extracted from the HIV database of the provincial health center (PHC). HIV-positive patients were identified by 2 sequential enzyme-linked immunosorbent assay tests positive for HIV antibodies, followed and confirmed by a western blot test. In Iran, PHCs are primarily responsible for registering and following patients with HIV/AIDS. The data on HIV/AIDS were reported to the PHC database from (a) transfusion center, (b) volunteer counseling and testing centers (VCTCs) affiliated with district health centers, and (c) prison. In Iran, blood transfusion center are parts of the national health system. Blood donation is voluntary, without payment. The costs of the collection, preparation, preservation, and distribution of blood and its components are supported by the government. All donated blood is tested for blood-borne diseases, including HIV [11]. VCTCs provide consulting and educational services to intravenous drug users (IDUs) in order to improve their knowledge of high-risk behaviors and harm reduction methods. Moreover, these centers provide diagnostic tests for IDUs, including testing for HIV, and refer individuals suspected to have HIV to specialized medical centers for medical care. Prisoners with a history of high-risk behaviors, such as IDUs and sex workers, are voluntarily tested for blood-borne infections, including HIV. Some HIV-positive patients were identified and registered in more than 1 data source. Nonetheless, none of these data sources had a complete list of HIV-positive individuals. We used the 3-source capture-recapture method to obtain a statistical estimate of the approximate number of HIV-positive individuals not identified by these data sources.

**Figure 1.**
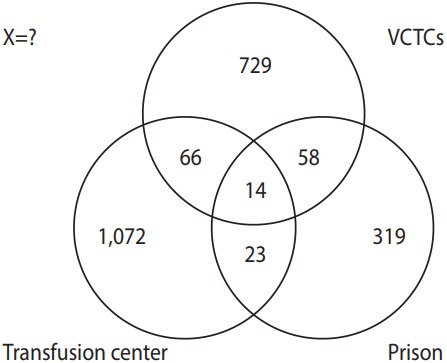
Distribution of the human immunodeficiency virus-positive patients across 3 data sources: transfusion center, volunteer counseling and testing centers (VCTCs), and prison. X, unregistered.

In order to perform a 3-source capture-recapture analysis, the lists of HIV-positive patients recorded in these 3 data sources were extracted and compared with each other to specify the common names listed in more than 1 data source. When an HIV-positive patient’s national identification code was not recorded in the data source, we used the patient’s demographic characteristics for comparison, including first name, second name, age, marital status, and residential area. We then arranged the data as shown in Figure 1.

In epidemiology, the capture-recapture approach attempts toestimate or adjust for the extent of incomplete ascertainment using information from overlapping lists of cases from different sources. This method provides an estimation of the affected population, and is particularly useful when the investigator has clearly incomplete data available from 2 or more sources [7]. In this study, we used the 3-source capture-recapture approach, including the 3 incomplete data sources of HIV-positive patients. The 3-source capture-recapture approach included the following 8 possible models:

(1) The number of HIV-positive patients identified by transfusion center only (A); (2) The number of HIV-positive patients identified by VCTCs only (B); (3) The number of HIV-positive patients identified by prison only (C); (4) The number of HIV-positive patients identified by A and B but not by C (AB); (5) The number of HIV-positive patients identified by A and C but not by B (AC); (6) The number of HIV-positive patients identified by B and C but not by A (BC); (7) The number of HIV-positive patients identified by all 3 sources (ABC); and (8) The number of HIV-positive patients identified by none of the three sources (X).

We applied the Poisson regression, or log-linear model, to accommodate the 3 sources of data, to explore the dependence among sources, and to adjust for dependence by including interaction terms in the model. For this purpose, we prepared a dataset with 4 variables, including: (a) variable *A*, with values of 0 or 1, which described belonging to list A; (b) variable *B*, with values of 0 or 1, which described belonging to list B; (c) variable *C*, with values of 0 or 1, that described belonging to list C; and (d) *freq*, which was a non-negative variable describing the frequency of observations in the combination of lists given by variables *A*, *B*, and *C*. The unknown frequency of cases occurring in none of the lists was considered to be missing. Based on the above available information, the missing value was estimated by the Poisson regression model.

We modeled dependence by using interaction terms. The absence of any third-order interaction (*ABC*= 0) is the basic assumption of the capture-recapture model [4]. By accommodating the 3 sources of data as described above, the log-linear model can estimate the number of HIV-positive patients not identified by any of the three centers (*X*), and thus the total population of HIV-positive patients (*N*).

We applied two different information criteria for model selection, including the Akaike information criterion (AIC) and the Bayesian information criterion (BIC) [12]. The AIC was calculated as follows:

(1) $$\mathrm{AIC}=G^{2}-\left[2\times \left(df\right)\right]$$

In equation (1), *G^2^* is the likelihood ratio statistic associated with the fit of any model to the data and *df* denotes the degrees of freedom of the model. The model with the smallest AIC value was selected. The second criterion, BIC, which is usually preferred to the AIC in some applications, was calculated as follows:

(2) $$\mathrm{BIC}=G^{2}-\left[\ln\left(N\mathrm{obs}/2\pi \right)\right]\times \left(df\right)$$

In equation (2), *G^2^* and *df* are defined as above, and ln(*N*obs) is the natural logarithm of the number of parameters in the model. As above, the model with the smallest BIC value was selected.

All analyses were performed at the 0.05 significance level using Stata version 14.0 (StataCorp., College Station, TX, USA).

## RESULTS

Of the 2,456 HIV-positive patients registered in the 3 data sources, 1,175 (47.8%) were identified by transfusion center, 867 (35.3%) by VCTCs, and 414 (16.8%) by prison. After duplicate entries were excluded, 2,281 HIV-positive patients remained. The characteristics of the study population by sources of data are given in Table 1. Most HIV-positive patients were single men aged 30-44 years. Most of the patients lived in urban areas and were unemployed. Injection drug use was the most common route of HIV transmission.

The results of the capture-recapture method are shown in Table 2. The p-values indicate that there were significant differences between the saturated model (the eighth model) and all other reduced models. The fifth model (ABC AB BC) was the best-fitting model, with the smallest AIC and BIC values. According to these results, it was estimated that about 14,868 (95% confidence interval [CI], 9,923 to 23,427) HIV-positive individuals were not identified by any of the data sources. Accordingly, the real number of HIV-positive individuals was estimated to be 17,149 (95% CI, 12,204 to 25,708).

The completeness of identifying HIV-positive patients by the 3 sources of data is shown in Table 3. Based on these findings, the completeness of the transfusion center, VCTCs, and prison was 6.9, 5.1, and 2.4%, respectively. Although, the completeness of the 3 sources of data was very low, the data from the transfusion center were more complete than the other sources.

## DISCUSSION

Estimating the number of HIV-positive individuals is essential for planning and providing health care services to this population. According to our estimation based on the capture-recapture method, many HIV-positive patients have not been identified and registered in any of the available sources of data. Some of these individuals may be unaware of their status and may play a role as a source of transmission of HIV among the population. This is a critical public health problem that requires special attention.

The completeness of these 3 sources of data was low. In addition, the proportion of overlapping information in the 3 sources was relatively low. Overlapping information plays an important role in estimating the missing population. Indeed, sufficiently high overlapping information is needed to produce a reliable estimate of the number of missing cases [4]. A relatively low overlap fraction is associated with a large number of singletons. In such cases, the missing population cannot be measured accurately due to insufficient overlap. Consequently, a large standard error is usually associated with the estimator in the equation [13]. Coull & Agresti [14] showed that the likelihood functions under some random effect models for low-overlap information might become flat, and that the estimated results based on equivalent log-linear models are likely to become unstable. This issue may explain the large standard error—and, hence, the wide CI—of the estimated number of unregistered HIV-positive individuals.

The p-values demonstrated a significant difference between the saturated and the reduced models. We applied the AIC and BIC to choose the best-fitting model. However, care must be taken when using AIC and BIC values for model selection. These criteria do not provide a test of a model in comparison with a null hypothesis. That means the AIC and BIC values say nothing about how well a model fits the data in an absolute sense. Therefore, if all possible log-linear models fit the data poorly, these values will not give any warning of the problem [15].

van Leth et al. [16] performed a 2-source capture-recapture analysis to estimate under-reporting in national databases of tuberculosis (TB) and HIV. According to the results of their study, the under-reporting of TB-HIV coinfection ranged from 50 to 70% in the national TB register. Héraud-Bousquet et al. [17] applied a 3-source capture-recapture method to estimate the number of new HIV diagnoses in children in France. They reported that the completeness of the 3 sources analyzed in their study was 28.4, 26.1, and 33.3%, respectively. The estimated completeness of the 3 sources combined was 55.8%. de Lemos et al. [18] estimated the number of HIV-positive pregnant women in Sergipe, Brazil, using a 3-source capture-recapture method. They reported that 381 (34.3%) pregnant women were not captured by any of the 3 systems.

The capture-recapture method is categorized into direct (2-sample) and indirect (multiple-sample) models. Although the direct model is time-consuming and difficult to implement in many cases, it provides better estimations. In contrast, implementing the indirect model is relatively simple and easy, but may not always result in an accurate estimate [19]. Although the indirect capturerecapture approach is a simple and attractive statistical approach for estimating the size of unknown and hard-to-reach populations, the results must be interpreted with caution due to the assumptions and limitations of the method. The capture-recapture method, like any other statistical procedure, has its assumptions and limitations. An important limitation of this method is that a sufficiently high overlap fraction is required to produce a reliable estimate of the missing population. Otherwise, the likelihood functions may become flat and the resulting estimates based on log-linear models may become unstable [14], as was the case in our study. Another critical assumption of the capture-recapture approach is the independence of the sources of data; otherwise, either positively or negatively dependent sources may result in underestimation or overestimation, respectively [5]. However, the log-linear model is able to handle dependence among sources of data and adjust for this dependence by including interaction terms in the model [4]. A critical limitation of the capture-recapture approach using log-linear models for estimating a missing population is that data sources with large sample sizes must satisfy the assumption of a normal distribution within log-linear models. If these assumptions are not considered, the estimates may not be reliable.

In conclusion, this study provided useful information about the unknown population of HIV-positive individuals based on the 3-source capture-recapture method. According to our findings, there are many HIV-positive individuals who have neither been diagnosed nor registered with any of the available sources of data. They themselves may be unaware of their status. This is a critical public health problem that should receive special attention from policymakers who plan and provide health care services for HIV-positive patients. However, the results of the capture-recapture method should be interpreted with caution due to its assumptions and limitations. These assumptions and limitations may lead to overestimation of the results as it seems possible in our results.

## Figures and Tables

**Table 1.** The distribution of the demographic characteristics of the HIV-positive patients registered with the 3 data sources

Characteristics	Transfusion center (n=1,175)	VCTCs (n=867)	Prison (n=414)
Sex			
Male	1,143 (97.3)	688 (79.3)	402 (97.1)
Female	32 (2.7)	179 (20.7)	12 (2.9)
Age (yr)			
<30	165 (14.0)	112 (12.9)	99 (23.9)
30-44	624 (53.1)	430 (49.6)	180 (43.5)
45-59	383 (32.6)	319 (36.8)	133 (32.1)
≥60	3 (0.3)	6 (0.7)	2 (0.5)
Marital status			
Married	286 (24.4)	298 (34.4)	212 (51.2)
Single	698 (59.4)	205 (23.6)	79 (19.1)
Divorced	99 (8.4)	238 (27.5)	89 (21.5)
Widowed	92 (7.8)	126 (14.5)	34 (8.2)
Residential area			
Urban	1,167 (99.3)	841 (97.0)	398 (96.1)
Rural	8 (0.7)	26 (3.0)	16 (3.9)
Occupational status			
Employee	7 (0.6)	16 (1.9)	1 (0.2)
Self-employed	480 (40.9)	227 (26.2)	68 (16.5)
Unemployed	688 (58.5)	624 (71.9)	345 (83.3)
Transmission			
Injection	789 (67.1)	622 (71.7)	298 (72.0)
Sexual	164 (14.0)	121 (14.0)	65 (15.7)
Vertical	0 (0.0)	13 (1.5)	1 (0.2)
Others	222 (18.9)	111 (12.8)	50 (12.1)

Values are presented as number (%).

HIV, human immunodeficiency virus; VCTCs, volunteer counseling and testing centers.

**Table 2.** Log-linear models fitted to the 3 lists of HIV-positive patients registered in the 3 data sources and the estimated number of unregistered HIV-positive individuals

Model	K	df	Log likelihood	G^2^	AIC	BIC	p-value	X	95% CI	
									Upper	Lower
ABC	3	3	-52.034	57.82	51.82	51.98	0.001	8,140	6,887	9,668
ABC AB	4	2	-28.082	9.92	5.92	6.02	0.01	11,511	9,362	14,294
ABC AC	4	2	-47.668	49.09	45.09	45.19	0.001	6,269	4,978	7,964
ABC BC	4	2	-50.395	54.54	50.54	50.65	0.001	7,470	6,192	9,068
ABC AB BC	5	1	-27.054	7.86	5.86	5.91	0.01	14,868	9,923	23,427
ABC AB AC	5	1	-28.012	9.78	7.78	7.83	0.001	11,841	9,205	15,477
ABC AC BC	5	1	-38.782	31.31	29.31	29.37	0.001	4,010	3,020	5,408
ABC AB BC AC	6	0	-23.125	0.00	0.00	0.00	1.00	39,641	18,145	84,600

HIV, human immunodeficiency virus; K, number of parameters; df, degree of freedom; G^2^ , deviance; AIC, Akaike information criterion; BIC, Bayesian information criterion; X, unregistered; CI, confidence interval.

**Table 3.** Completeness of the detection of HIV-positive individuals using the 3 registers

Registry	n	Completeness (95% CI, %)
Transfusion center	1175	6.9 (4.6,9.6)
VCTCs	867	5.1 (3.4, 7.1)
Prisons	414	2.4 (1.6, 3.4)
Total, excluding duplicates^1^	2,281	13.3 (8.9,18.7)

HIV, human immunodeficiency virus; CI, confidence interval; VCTCs, volunteer counseling and testing centers.

1 Considering an estimated number of unregistered HIV-positive individuals of 14,868, and a total estimated number of HIV-positive patients after excluding duplicates of 17,149 (95% CI, 12,204 to 25,708).

## References

[1] World Health Organization (2010). Guidelines on estimating the size of populations most at risk to HIV. http://data.unaids.org/pub/manual/2010/guidelines_popnestimationsize_en.pdf.

[2] Bernard HR, Johnsen EC, Killworth PD, Robinson S (1991). Estimating the size of an average personal network and of an event subpopulation: some empirical results. Soc Sci Res.

[3] Clague A (2001). Introductory note on census enumeration. https://unstats.un.org/unsd/demographic/meetings/egm/symposium2001/docs/symposium_09.htm.

[4] Chao A, Tsay PK, Lin SH, Shau WY, Chao DY (2001). The applications of capture‐recapture models to epidemiological data. Stat Med.

[5] University of Pittsburgh (2017). Capture-recapture. http://www.pitt.edu/~yuc2/cr/.

[6] Poorolajal J, Haghdoost AA, Mahmoodi M, Majdzadeh R, Nasseri-Moghaddam S, Fotouhi A (2010). Capture-recapture method for assessing publication bias. J Res Med Sci.

[7] Hook EB, Regal RR (1995). Capture-recapture methods in epidemiology: methods and limitations. Epidemiol Rev.

[8] Khazaei S, Poorolajal J, Mahjub H, Esmailnasab N, Mirzaei M (2012). Estimation of the frequency of intravenous drug users in Hamadan city, Iran, using the capture-recapture method. Epidemiol Health.

[9] Amin-Esmaili M, Nedjat S, Motevalian A, Rahimi-Movaghar A, Majdzadeh R (2009). Comparison of databases for Iranian articles; access to evidence on substance abuse and addiction. Arch Iran Med.

[10] Karami M, Khazaei S, Poorolajal J, Soltanian A, Sajadipoor M (2017). Estimating the population size of female sex worker population in Tehran, Iran: application of direct capture-recapture method. AIDS Behav.

[11] Cheraghali A (2012). Overview of blood transfusion system of iran: 2002-2011. Iran J Public Health.

[12] Hook EB, Regal RR (1997). Validity of methods for model selection, weighting for model uncertainty, and small sample adjustment in capture-recapture estimation. Am J Epidemiol.

[13] Seber GA (1992). A review of estimating animal abundance II. Int Stat Rev.

[14] Coull BA, Agresti A (1999). The use of mixed logit models to reflect heterogeneity in capture‐recapture studies. Biometrics.

[15] Wikipedia Akaike information criterion. https://en.wikipedia.org/wiki/Akaike_information_criterion,_27_March_2017.

[16] van Leth F, Evenblij K, Wit F, Kiers A, Sprenger H, Verhagen M (2016). TB-HIV co-infection in the Netherlands: estimating prevalence and under-reporting in national registration databases using a capture-recapture analysis. J Epidemiol Community Health.

[17] Héraud-Bousquet V, Lot F, Esvan M, Cazein F, Laurent C, Warszawski J (2012). A three-source capture-recapture estimate of the number of new HIV diagnoses in children in France from 2003-2006 with multiple imputation of a variable of heterogeneous catchability. BMC Infect Dis.

[18] de Lemos LM, Duarte GS, Martins NG, da Silva FJ, Ilozue C, Gurgel RQ (2013). Estimating the number of HIV-positive pregnant women in Sergipe, Brazil, using capture-recapture. AIDS Care.

[19] Posbic KE (2010). Comparison of direct and indirect methods of estimating migration and dispersal routes of the marbled salamander, Ambystoma opacum.

